# Surface functionalization of 3D-printed plastics via initiated chemical vapor deposition

**DOI:** 10.3762/bjnano.8.162

**Published:** 2017-08-08

**Authors:** Christine Cheng, Malancha Gupta

**Affiliations:** 1 Mork Family Department of Chemical Engineering and Material Science, University of Southern California, 925 Bloom Walk, Los Angeles, California 90089, USA

**Keywords:** 3D printing, chemical vapor deposition, coatings, functional polymers, surface modification

## Abstract

3D printing is a useful fabrication technique because it offers design flexibility and rapid prototyping. The ability to functionalize the surfaces of 3D-printed objects allows the bulk properties, such as material strength or printability, to be chosen separately from surface properties, which is critical to expanding the breadth of 3D printing applications. In this work, we studied the ability of the initiated chemical vapor deposition (iCVD) process to coat 3D-printed shapes composed of poly(lactic acid) and acrylonitrile butadiene styrene. The thermally insulating properties of 3D-printed plastics pose a challenge to the iCVD process due to large thermal gradients along the structures during processing. In this study, processing parameters such as the substrate temperature and the filament temperature were systematically varied to understand how these parameters affect the uniformity of the coatings along the 3D-printed objects. The 3D-printed objects were coated with both hydrophobic and hydrophilic polymers. Contact angle goniometry and X-ray photoelectron spectroscopy were used to characterize the functionalized surfaces. Our results can enable the use of iCVD to functionalize 3D-printed materials for a range of applications such as tissue scaffolds and microfluidics.

## Introduction

Three-dimensional printing (3DP) is a useful fabrication technique that offers rapid and low-cost prototyping, high levels of design complexity, and resolution on the micron scale [1–2]. These attractive features have led to applications of 3DP in diverse fields including tissue engineering [2–3], microfluidics [4], robotics [5], and batteries [6–7]. 3DP involves a computer-aided design of the target structure sliced into 2D layers and printed layer-by-layer [2–3]. Four methods of 3DP are most common. Fused deposition modeling (FDM) involves heating a feed filament past the melting point of the material and extruding it onto a platform, which moves progressively downwards as layers are printed [8–9]. Inkjet printing deposits droplets of ink onto a platform, with ink flow regulated by a piezoelectric actuator [10–11]. Selective laser sintering uses a laser beam to heat a layer of powder above its melting point, fusing it to the previous layers, and then new powder is subsequently rolled over the printed object [12–13]. In stereolithography (SLA), a laser or UV beam selectively hardens layers of photocurable resin and then the object is covered with another layer of fresh resin [14–15].

Though the number of printable functional materials is burgeoning [1,16–17], tuning the materials properties within the constraints of printability is still a challenge. This limitation presents a problem for application-driven print objects, because consideration of material printability must supersede other functionalities, such as biocompatibility or responsiveness to stimuli. Thus, controlling post-printing surface properties is critical to expanding the breadth of 3DP applications, because it allows for tuning of bulk properties, such as cost-effectiveness or structural rigidity, independently of sophisticated surface functionalization. For example, in scaffolds for bone tissue engineering, angiogenesis is a major challenge, because printed scaffolds have hydrophobic surface properties and do not promote cellular differentiation [3,18]. Surface modification of printed scaffolds can allow for the tuning of surface functionalization to promote vascularization and tissue regeneration while maintaining control over the mechanical robustness of the bulk structure. Hong et al. demonstrated that simply dipping polycaprolactone/poly(lactic-*co*-glycolic acid) 3D scaffolds in mussel adhesive proteins promoted cellular adhesion, proliferation and differentiation, showing that a facile surface modification improved the viability of using 3D-printed scaffolds for tissue engineering applications [18]. In another example of surface functionalization, Wang et al. reported a method for modifying the surfaces of 3DP structures fabricated via SLA by using a UV-curable resin with an embedded alkyl bromide initiator from which atom transfer radical polymerization was initiated [19–20]. They demonstrated that complex 3D-printed structures could be coated with hydrophobic polymers and various metals. However, this coating technique is limited to photocurable resins into which the polymerization initiator has already been incorporated, which restricts surface modification to only SLA-printed objects and wastes unused initiator embedded within the bulk structure. The breadth of materials and feature sizes of 3D-printed objects presents a challenge to finding a universal method for surface functionalization.

Initiated chemical vapor deposition (iCVD) is a technique that can be used to deposit functional polymer coatings [21–22]. In the iCVD process, monomer and *tert*-butyl peroxide (TBPO) initiator are introduced in the vapor phase to a reactor chamber under vacuum, whereupon the initiator is thermally cleaved by a heated filament array. Monomer and initiator radicals adsorb to substrates on a cooled stage where polymerization occurs. The molecular weight increases with decreasing substrate temperature and typical molecular weights are in the range of 50,000 to 200,000 [23–24]. The iCVD process is solventless and therefore effects of surface tension are avoided, allowing for conformal coating on complex surfaces such as mictrotrenches [25] and nanopore membranes [26]. Since the rate of reaction in iCVD is limited by adsorption of monomer to the substrate, a lower substrate temperature results in a faster polymerization rate [24]. Thus, the thermally insulating properties of macro-scale 3D-printed plastics pose a challenge to the iCVD process. Although there have been previous reports of iCVD deposition onto thermally insulating substrates such as tissue wipes [27], glass [28], and poly(ethylene naphthalate) [29], these substrates were typically less than 1 mm in thickness and therefore the thermal gradients were modest. In contrast to these previous studies, our 3D-printed objects are over 5 mm in thickness and therefore the significant thermal gradients may impact the deposition process.

In this study, we printed the 3D objects using both poly(lactic acid) (PLA) and acrylonitrile butadiene styrene (ABS) in order to study the generality of the coating process for modifying the surfaces of different plastics. We investigated the deposition of poly(1*H*,1*H*,2*H*,2*H*-perfluorodecyl acrylate) (PPFDA) [23] and poly((2-hydroxyethyl methacrylate)-*co*-(ethylene glycol diacrylate)) (P(HEMA-*co*-EGDA)) [30] onto 3D objects of a variety of shapes and sizes to study the capabilities and limitations of the iCVD process. X-ray photoelectron spectroscopy (XPS) and contact angle goniometry were used to study the surface properties before and after coating.

## Results and Discussion

**Figure 1:**
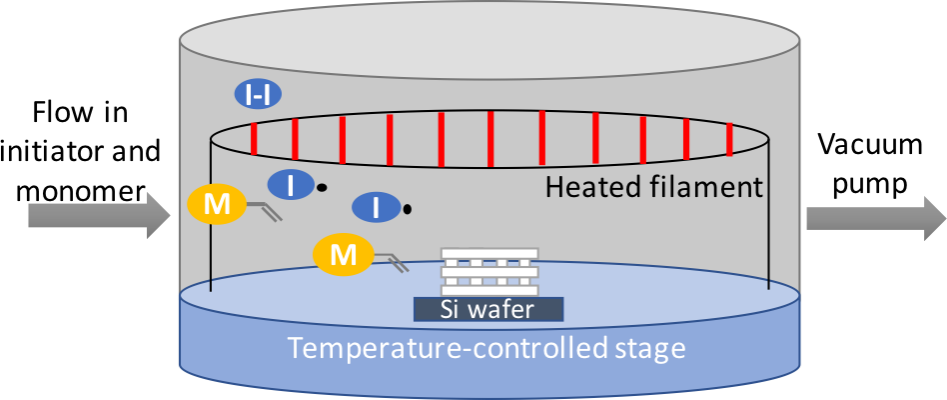
Schematic of the iCVD process. The 3D-printed substrate (white lattice) is placed on a silicon wafer piece on a temperature-controlled stage. Initiator (I–I) and monomer (M) in vapor phase are introduced into the reactor and the filament array is heated to thermally cleave the initiator.

A schematic of the iCVD deposition process onto 3D-printed substrates is shown in Figure 1. To systematically study the uniformity of the iCVD coatings, PPFDA was deposited onto 3D-printed PLA lattices of 7.5 mm in height. PPFDA was chosen as a model polymer because it is easily discernable from the underlying substrate via XPS [23]. Additionally, the relatively high water contact angle on flat PPFDA (121°) [31] compared to that on flat PLA (72.5°) [32] allows for the use of contact angle goniometry to verify polymer deposition. Substrates were printed with PLA because of its ease of printing, low cost, and prior use in biomedical applications [33]. A silicon wafer piece was placed under the substrate to visually observe the penetration of polymer through the lattice.

**Figure 2:**
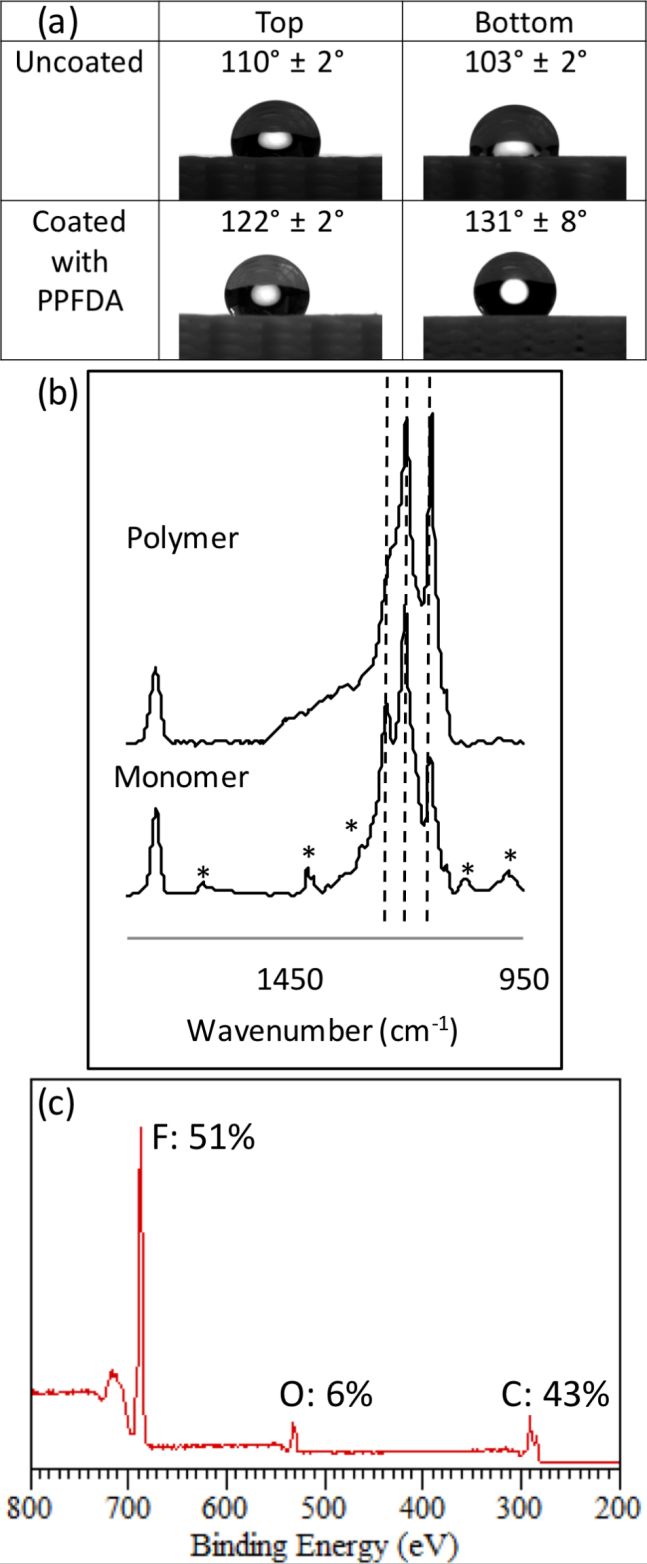
(a) Static contact angles for a 7.5 mm tall PLA lattice that was uncoated and coated with PPFDA. (b) FTIR spectra of the PPFDA film (top) and liquid monomer (bottom). Dashed lines correspond to the CF_2_ and CF_3_ signals, and the asterisks in the monomer spectrum correspond to the vinyl signals. (c) Representative XPS survey spectrum of the top of the lattice coated with PPFDA.

**Figure 3:**
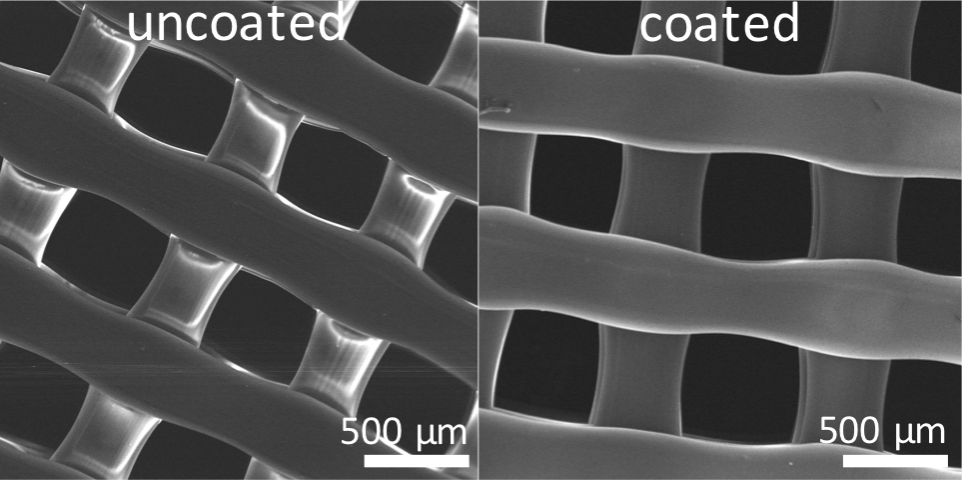
SEM images of the lattice before (left) and after (right) PPFDA coating.

To measure the change in hydrophobicity of the 7.5 mm PLA lattice after the deposition of PPFDA, contact angle changes were monitored (Figure 2a). Variations in contact angle measurements at the top and bottom of the pieces and among different pieces can be attributed to slight variations in geometry during the printing process. During deposition, the top side was closer to the heated filament array and the bottom side was placed on a silicon wafer piece on the stage. After coating, the contact angle changed from (110 ± 2)° to (122 ± 2)° at the top of the lattice and from (103 ± 2)° to (131 ± 8)° at the bottom, indicating that both the top and bottom of the lattice were coated with PPFDA. The contact angles are higher than that of flat PLA and flat PPFDA due to surface roughness [34]. Penetration of polymer through the lattice was also confirmed by deposition on the silicon wafer piece underneath the lattice. We used Fourier transform infrared spectroscopy (FTIR) (Figure 2b) to compare the spectra of the PPFDA film deposited on the silicon wafer (top) and the liquid monomer (bottom). The peaks at 1250, 1200, and 1150 cm^−1^ in the polymer confirm the presence of the CF_2_ and CF_3_ groups and the absence of signals from the vinyl bond in the polymer spectrum at 1640, 1620, 1410, 1400, 1300, 1080, 986, and 971 cm^−1^ indicates that all the vinyl bonds were completely reacted. Additionally, the presence of PPFDA at the top of the lattice was verified using X-ray photoelectron spectroscopy (XPS) to analyze the chemical composition of the surface (Figure 2c). The survey spectrum of the top of the PPFDA-coated lattice had atomic percentages of 51.2 atom % F, 6.2 atom % O, and 42.6 atom % C on a hydrogen-free basis, which agreed well with the theoretical composition of PPFDA (53.1 atom % F, 6.3 atom % O, 40.6 atom % C) rather than that of PLA (40 atom % O, 60 atom % C) indicating that there is at least 5 nm of PPFDA coating at the top of the lattice since XPS probes the top 5 nm of the surface. Scanning electron microscopy (SEM) images of the lattice (Figure 3) reveal that the appearance before modification (left) and after PPFDA coating (right) are similar since the thickness of the polymer coating is much smaller than the feature size.

The stage temperature can impact the thermal gradient during polymerization. The concentration of monomer at the surface of the substrate increases with decreasing temperature as previously shown by quartz crystal microbalance experiments by Lau and Gleason [24]. At a given stage temperature, we expect different temperatures at the top and bottom of the 3D-printed objects due to the heat from the filament array. To systematically study this effect, we studied depositions at stage temperatures of 15 °C, 35 °C and 45 °C (Table 1). For these stage temperatures, the temperature at the bottom of a 7.5 mm lattice was measured to be 31 °C, 43 °C and 48 °C, respectively, and the temperature at the top of the lattice was measured to be 62 °C, 77 °C and 80 °C, respectively. These temperature differences of ca. 30 °C are due to the large height and thermally insulating properties of the PLA lattice. After PPFDA deposition at a stage temperature of 15 °C (sample S1), the contact angle of the lattice increased from (105 ± 2)° to (126 ± 5)° at the top and from (99 ± 8)° to (131 ± 4)° at the bottom. After PPFDA deposition at a stage temperature of 35 °C (sample S2), the contact angle at the top of the lattice increased from (109 ± 5)° to (127 ± 3)° at the top and from (104 ± 2)° to (139 ± 3)° at the bottom. After PPFDA deposition at a stage temperature of 45 °C (sample S3), the contact angle of the lattice increased from (111 ± 6)° to (125 ± 4)° at the top and from (105 ± 2)° to (133 ± 4)° at the bottom. These increases of the contact angle indicate that the lattices were coated at both the top and bottom at the three stage temperatures, despite the large temperature gradients. To further verify the presence of the PPFDA coatings, XPS was used to measure the atomic composition at the top and bottom of the lattices (Table 1). For the three stage temperatures, the atomic compositions of the bottom match well with the theoretical composition of PPFDA, again indicating that there is at least 5 nm of coating at the bottom of the lattice, however the top sides of S1 and S2 have slightly less fluorine indicating less coating.

**Table 1:** XPS survey spectra for PLA lattices.

sample	stage temperature (°C)	filament temperature (°C)	position	atom % F	atom % O	atom % C

reference PPFDA				53.1	6.3	40.6
S1	15	250	top	43.8	8.1	48.1
			bottom	49.6	6.6	43.8
S2	35	250	top	46.4	6.6	47.0
			bottom	51.1	6.2	42.7
S3	45	250	top	53.0	7.0	40.0
			bottom	51.6	6.2	42.2
F1	35	220	top	50.7	6.9	42.4
			bottom	50.1	6.5	43.4
F2	35	190	top	49.7	6.8	43.5
			bottom	53.9	6.3	39.8
H1	15	250	top	27.9	15.7	56.4
			bottom	52.9	6.4	40.7
H2	45	250	top	43.8	7.9	48.3
			bottom	53.3	6.5	40.2
absorption	45	250	top	19.0	15.3	65.7
			bottom	26.2	13.8	60.0

Another challenge for iCVD deposition onto plastic materials is the potential for precursor molecules to absorb into the substrate. To verify that our XPS signals are due to polymer and not due to monomer, a PLA lattice was placed in the reactor and exposed to the same deposition conditions except without the presence of initiator. Polymerization does not occur because of the absence of the initiator, but the heated filament causes the same thermal gradients in the PLA lattice that were present during depositions. After monomer exposure, the contact angle changed from (106 ± 3)° to (119 ± 5)° at the top of the lattice and from (107 ± 4)° to (119 ± 4)° at the bottom of the lattice. This contact-angle increase indicates that some monomer was absorbed into the lattice. XPS of the sample (Table 1) shows the presence of a fluorine signal, which is absent in PLA, confirming the presence of monomer in the lattice. However, this fluorine signal is much less than that for the PPFDA polymer. Therefore, we can conclude that although there may be some monomer absorption during deposition, the large fluorine signals from the samples S1–S3 match the theoretical PPFDA values and therefore confirm the presence of a polymer coating of more than 5 nm.

To decrease thermal gradients during the deposition of PPFDA onto the PLA lattices, the filament temperature can be reduced. We therefore investigated whether a uniform coating could still be achieved with lower filament temperatures. The filament temperature was lowered to 220 °C (sample F1) and the contact angles changed from (106 ± 5)° to (127 ± 3)° at the top of the lattice and from (102 ± 6)° to (140 ± 3)° at the bottom of the lattice. The filament temperature was further lowered to 190 °C (sample F2) and the contact angles changed from (106 ± 3)° to (128 ± 2)° at the top of the lattice and from (99 ± 6)° to (133 ± 5)° at the bottom of the lattice. At both filament temperatures, the top and bottom of the lattice exhibited increasing contact angles, indicating that the lattices could be coated at lower filament temperatures. Additionally, XPS of the lattices (Table 1) showed that the atomic composition agreed well with that of PPFDA, indicating that there is at least 5 nm of PPFDA at the top and bottom of both samples F1 and F2.

To further investigate the effects of thermal gradients, the lattice size was increased to 25 mm, which reaches to 6 mm below the filament array. For stage temperatures of 15 °C and 45 °C, the temperature at the top of the lattice was measured to be 97 °C and 103 °C, respectively. At a stage temperature of 15 °C (sample H1), the contact angle changed from (107 ± 5)° to (118 ± 4)° at the top of the lattice and from (103 ± 8)° to (125 ± 5)° at the bottom of the lattice. XPS of the sample (Table 1) showed that the atomic composition at the bottom agreed well with PPFDA, however, the composition at the top was similar to the signal found for monomer absorption, indicating that deposition did not occur. For a stage temperature of 45 °C (sample H2), the contact angle changed from (101 ± 3)° to (123 ± 2)° at the top of the lattice and from (93 ± 7)° to (134 ± 6)° at the bottom of the lattice. XPS of the sample (Table 1) showed that the bottom was coated, however, the decreased fluorine coating at the top demonstrated less coating. These samples indicate that the thermal gradients in very tall 3D objects can inhibit polymerization close to the filament. These thermal effects could be combatted by increasing the height of the filament array, optimizing substrate orientation, or lowering the substrate temperature.

**Figure 4:**
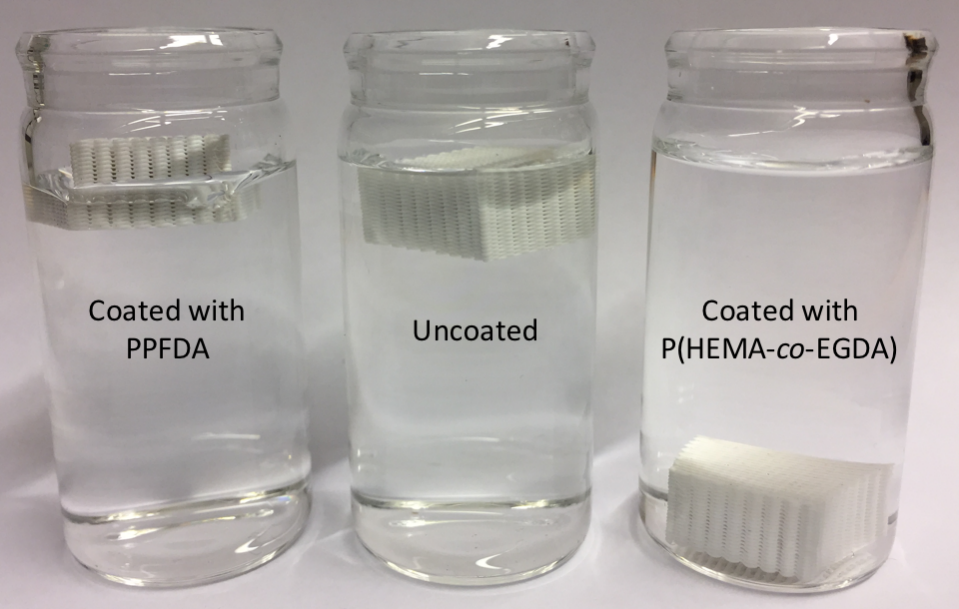
PLA lattices coated with PPFDA (left), uncoated (center), and coated with P(HEMA-*co*-EGDA) (right) in water.

To demonstrate the generality of the iCVD process for depositing different functional polymers onto 3D-printed substrates, 7.5 mm tall lattices were also coated with a hydrophilic, cross-linked polymer. PHEMA was chosen as the model hydrophilic polymer because it is biocompatible and has been used in biomedical applications [35–36]. However, because PHEMA is water soluble, the cross-linker EGDA was incorporated during the deposition to ensure that the hydrophilic polymer coating would not dissolve in water. As shown in Figure 4, an uncoated PLA lattice did not sink in water, despite PLA having a density of 1.25 g/cm^3^. Since the uncoated PLA is hydrophobic, the pores of the lattice remained filled with air instead of wetting readily, which sufficiently reduced the overall density of the lattice causing it to float. Similarly, a lattice coated with PPFDA did not sink, because its enhanced hydrophobicity caused its pores to also remain filled with air. Unlike the hydrophobic lattices, the lattice coated with P(HEMA-*co*-EGDA) wicked water into its pores because of the hydrophilicity and the lattice sank in the water. To demonstrate the efficacy of the incorporated cross-linker for preventing dissolution of the polymer coating, the lattice coated with P(HEMA-*co*-EGDA) was soaked in water for three days and then was dried and placed back into the water, whereupon it sank, demonstrating the retention of its hydrophilicity.

**Figure 5:**
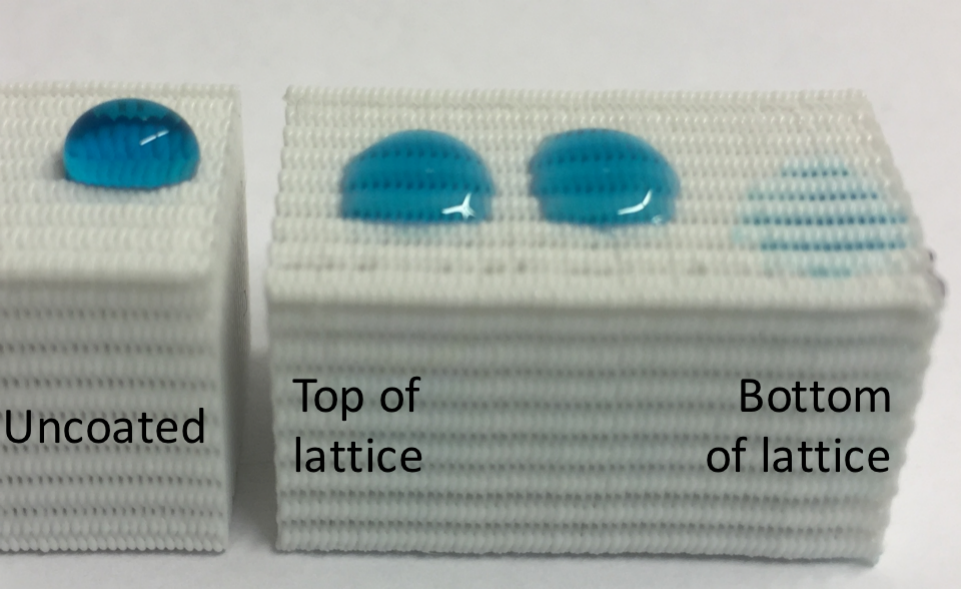
Water droplets (colored with blue food coloring) on an uncoated lattice and on a 25 mm tall PLA lattice coated with P(HEMA-*co*-EGDA).

To study the limitations of the iCVD process for coating macro-scale plastics, P(HEMA-*co*-EGDA) was deposited onto a 25 mm tall lattice. In Figure 5, the bottom of the lattice was placed on the silicon wafer piece on the stage and the top of the lattice was nearest to the filament. From Figure 5, the bottom of the lattice exhibited hydrophilic properties verifying that it was coated with P(HEMA-*co*-EGDA). The middle and top of the lattice wicked water, but less readily than the bottom of the lattice, indicating partial polymer coverage. Comparison of the droplets on the coated lattice show that the coated lattice is more hydrophilic than the uncoated lattice.

**Figure 6:**
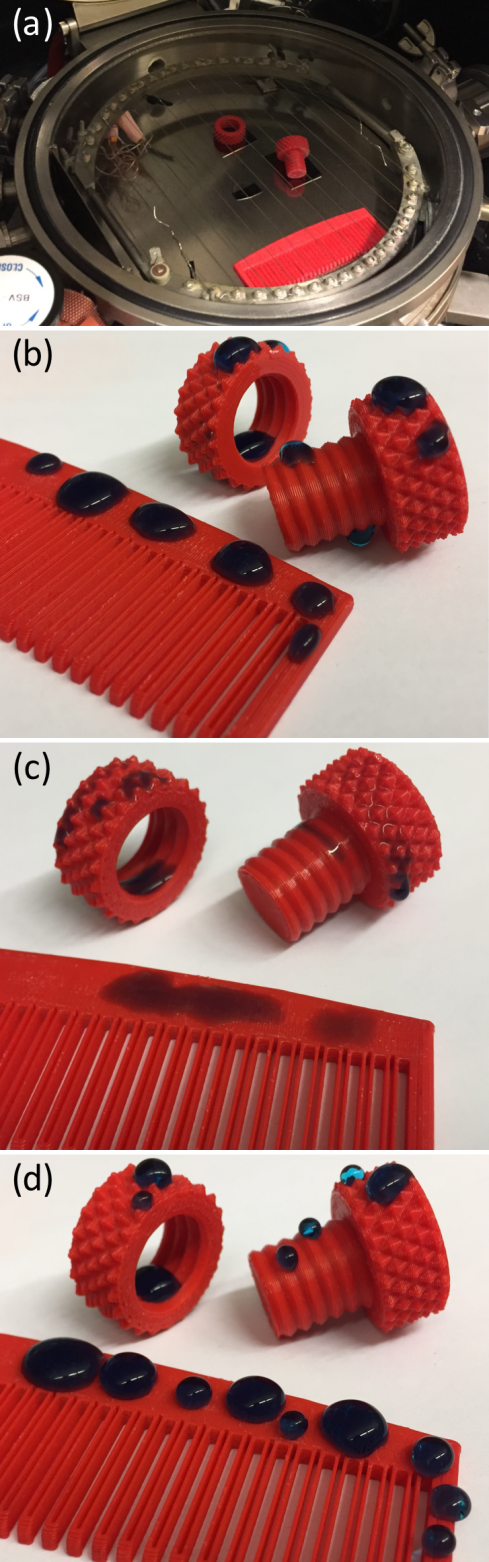
Sequential deposition of P(HEMA-*co*-EGDA) and PPFDA. (a) Nut, bolt, and comb in the iCVD reactor. Water droplets (colored with blue food coloring) on ABS substrates (b) without coating, (c) coated with P(HEMA-*co*-EGDA), and (d) coated with PPFDA.

A unique feature of the iCVD process is facile layering of polymer coatings with different chemistries, which allows for tuning of surface properties. To demonstrate this feature, substrates were coated with a hydrophilic copolymer followed by a hydrophobic polymer. Substrates were printed with ABS to demonstrate the generality of the substrate material. A comb, nut, and bolt were all coated in the same deposition to show that objects with complex features can be easily coated using the iCVD process (Figure 6a). The uncoated ABS substrate surfaces were hydrophobic (Figure 6b). The substrates were first coated with P(HEMA-*co*-EGDA), after which the comb, nut, and bolt all were readily wetted (Figure 6c). Following the coating with hydrophilic polymer, the substrates were then coated with PPFDA, and the substrate surfaces regain hydrophobicity (Figure 6d). These sequential depositions of polymers with different chemistries show that the substrate surface properties can be readily tuned using the iCVD process.

## Conclusion

The iCVD process was used to modify the surfaces of 3D-printed polymer substrates with complex geometries. The lattices studied were 7.5 mm and 25 mm tall, which were significantly larger than insulating substrates that were coated in previous iCVD reports. Both hydrophobic (PPFDA) and hydrophilic (P(HEMA-*co*-EGDA)) polymer coatings were deposited onto substrates made of PLA and ABS. Thermal gradients over PLA lattices were studied and shown to decrease polymer coverage on 25 mm substrates, but these effects can be overcome by optimizing substrate orientation and lowering the substrate temperature. Additionally, the surface properties of the substrates can be tuned using sequential polymer depositions. This coating process can be generalized to modify the surface properties of a variety of 3D-printed materials for potential applications in tissue grafting, microfluidics, and electronics.

## Experimental

1*H*,1*H*,2*H*,2*H*-Perfluorodecyl acrylate (PFDA) (SynQuest Laboratories, 97%), 2-hydroxyethyl methacrylate (HEMA) (Aldrich, 97%), ethylene glycol diacrylate (EGDA) (Polysciences, Inc.), and *tert*-butyl peroxide (TBPO) (Aldrich, 98%), were used as received without further purification. 3D-printed PLA lattices (Invention’s Hub, Mission Hills, CA) were also used as received. The comb, nut, and bolt were printed on a MakerBot Replicator 2X using ABS filament in True Red.

Polymerization was carried out in a custom-built iCVD reactor (GVD Corporation, 250 mm diameter, 48 mm height). Substrates were placed on silicon wafer pieces (Wafer World, 100 mm) on a stage that was temperature-controlled by a backside recirculating chiller (Thermo Scientific NESLAB RTE 7). The orientation of the lattice during polymer deposition was controlled for consistency. In FDM, the first layer is printed onto a heated, flat build plate which causes the bottom of the layer to be discernably flatter than subsequent extruded layers. This first printed layer was placed facing downwards during all iCVD depositions. Prior to polymer deposition, the stage was cooled to 14 °C for an hour to reduce the temperature of the substrates. Reactor pressure was achieved by a rotary vane vacuum pump (Edwards E2M40) controlled by a throttle valve (MKS 153D) and measured with an ambient temperature capacitance manometer (MKS 622C01TDE Baratron). Monomers were loaded into stainless steel jars and subsequently attached to the reactor chamber. To achieve appropriate monomer vapor pressure, PFDA was heated to 60 °C or 50 °C (for S1-S3, F1-F2, H1-H2), HEMA was heated to 50 °C, and EGDA was heated to 35 °C. TBPO was kept at room temperature of 25 °C and introduced into the reactor using a mass flow controller (MKS Type 1152C).

Immediately before polymer deposition, the stage temperature was raised to the appropriate temperature for deposition, which was 35 °C unless otherwise stated. During deposition, a nichrome filament (Omega Engineering, 80%/20% Ni/Cr) array held at 31 mm above the substrates was resistively heated to 250 °C, unless otherwise stated, to thermally cleave the peroxide bond of the initiator. The deposition rate was monitored in situ via interferometry on a reference silicon wafer using a He–Ne laser (Industrial Fiber Optics, 633 nm). For the samples S1–S3, F1–F2 and H1–H2, PFDA and TBPO were introduced into the reactor at flow rates of 0.26 sccm and 1.8 sccm, respectively. Reactor pressure was maintained at 50 mTorr, and deposition was carried out for 1 h. For the other depositions of the hydrophobic coating, PFDA and TBPO were introduced into the reactor at flow rates of 0.6 sccm and 1.0 sccm, respectively. Reactor pressure was maintained at 100 mTorr and deposition proceeded for 1 h. For the deposition of the cross-linked hydrophilic coating, HEMA was introduced at a flow rate of 1.0 sccm, EGDA was introduced at 0.14 sccm, and TBPO was introduced at 1.3 sccm. Reactor pressure was maintained at 130 mTorr and deposition was carried out for 1.5 h. To coat samples with multiple polymer layers, the substrates were removed from the reactor, rinsed with deionized water, and characterized between polymer layers.

Contact angles were measured on a goniometer (ramé-hart 290) with 10 μL droplets of deionized water. Five measurements were taken per sample and averaged and profile images were taken using the goniometer camera. Additionally, because the lattice structure consists of alternating, crosshatched layers, the structure has visible grooves depending on the viewing orientation. Therefore, to measure contact angles, the lattice was oriented such that the grooves were orthogonal to the goniometer camera. The chemical functionality of samples was studied using a Fourier transform infrared spectrometer (Thermo Scientific i510), with a resolution of 4 scans over a total of 32 scans. The surface composition of samples was studied using X-ray photoelectron spectrometer (Kratos Axis Ultra DLD) with a monochromatic Al Kα source. Survey spectra were taken from 0 to 800 eV in steps of 1 eV, averaged over five scans. Sample morphology was studied using a scanning electron microscope (Topcon Aquila), and samples were sputtered with a thin layer of silver (Cressington Sputter Coater 108) prior to imaging.
